# Peritumoral Immunoglobulin M Lambda Light Chain Amyloidosis in a Patient With Advanced Follicular Lymphoma

**DOI:** 10.7759/cureus.21738

**Published:** 2022-01-30

**Authors:** Kalpesh Shah, Sudarsan v Kollimuttathuillam, Nyan Bethel, Hamid Shaaban

**Affiliations:** 1 Internal Medicine, Saint Michael's Medical Center, Newark, USA; 2 Oncology, Saint Michael's Medical Center, Newark, USA; 3 Hematology/Oncology, Saint Michael's Medical Center, Newark, USA

**Keywords:** peritumoral, amyloidosis, light chain, lymphoma, follicular

## Abstract

Peritumoral light chain (AL) amyloidosis secondary to lymphoid malignancies is a rare but well-described entity. Peritumoral deposition of amyloid without systemic amyloidosis has been described in mucosa-associated lymphoid tissue (MALT) lymphomas; however, there are no reported cases of follicular lymphoma with localized peritumoral AL amyloidosis without systemic involvement of amyloidosis. We present a rare case of a patient with advanced follicular lymphoma with peritumoral lymph node IgM lambda light chain amyloidosis without an underlying monoclonal gammopathy or plasma cell dyscrasia.

## Introduction

Light chain (AL) amyloidosis is a rare disorder usually secondary to plasma cell dyscrasia (“small dangerous clone of cells”) and less commonly clonal lymphoproliferative disorders [1,2]. This hematological disorder is typically associated with hematologic malignancies like plasma cell dyscrasias (multiple myeloma, primary systemic amyloidosis, and plasmacytoma) and lymphoproliferative disorders (chronic lymphocytic leukemia, lymphoplasmacytic lymphoma including Waldenstrom macroglobulinemia, and marginal zone lymphoma) which result in an overproduction of immunoglobulin chains [3]. The clinical spectrum of immunoglobulin-derived amyloidosis may range between systemic involvement with end-organ involvement with amyloidosis and the peritumoral form of the disorder in which the amyloid remains at the site of production [4]. Peritumoral types of AL amyloidosis may involve the lower urinary tract [5], lungs [6,7], head and neck [8,9], and gastrointestinal tract [10,11]. In 2-5% of AL amyloidosis, an underlying B cell lymphoproliferative disorder may be found [12,13]. The lymphoproliferative disorders that have been described in the medical literature to date include lymphoplasmacytic lymphoma, chronic lymphocytic leukemia, and marginal zone lymphoma [14-17]. Typically, in the cases associated with peritumoral AL amyloidosis, patients have an absent to low level of monoclonal protein. Herein, we present the first reported case of a patient with advanced follicular lymphoma associated with peritumoral IgM lambda amyloidosis without a monoclonal protein component on serum and urine immunofixation studies.

## Case presentation

**Figure 1:**
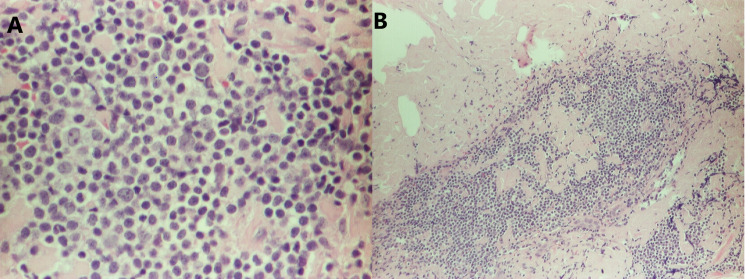
A (left) reveals H&E staining for centroblasts and centrocytes, and B (right) reveals H&E staining revealing amyloid

**Figure 2:**
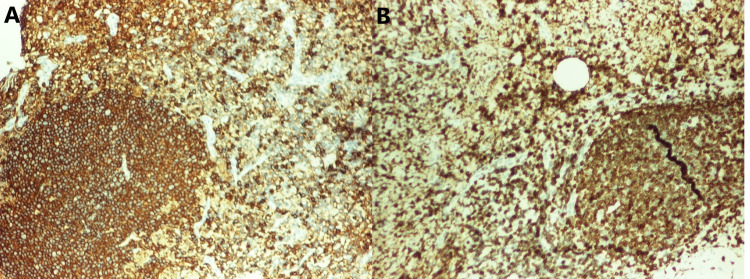
A (left) reveals positive staining for CD20, and B (right) reveals positive staining for BCL2

**Figure 3:**
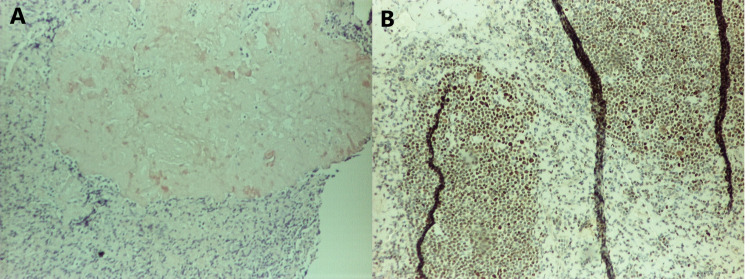
A (left) reveals positive staining for Congo red stain, and B (right) reveals positive staining for BCL6

A sixty-three-year-old African-American man with a past medical history of anemia and schizophrenia presented to the clinic with complaints of fever, night sweats, and generalized lymphadenopathy. Diffuse lymphadenopathy and massive splenomegaly were visualized on his CT of the chest, abdomen, and pelvis. He underwent an excisional right cervical lymph node biopsy. On histopathological examination of lymph node, the nodal architecture was effaced by nodular lymphoid proliferation in a background of abundant pink, amorphous substance. The follicles were poorly defined and showed a lack of polarization and tingible body macrophages. They were composed mostly of centrocytes and centroblasts (grade 3 follicular lymphoma), accounting for 6-15/HPF (Figure 1). The immunohistochemical study showed the cells to be positive for CD20, PAX5, CD10, BCL2, and BCL6 (Figure 2). CD21 stain was positive, which highlighted the follicular dendritic cell meshworks. Plasma cells expressed monoclonal IgM lambda. Congo red stain (Figure 3) was positive for amyloid deposition.

The findings correlated with a diagnosis of follicular lymphoma with plasmacytic differentiation and peritumoral IgM lambda AL amyloid deposition. The immunohistochemical study was negative for MYD88. Serum and urine immunofixation and electrophoresis studies were negative for any plasma cell dyscrasia or monoclonal gammopathy. The patient had a bone marrow biopsy, and the histopathological examination showed marrow involvement of monoclonal lambda restricted B cell population (30%) suggestive of follicular lymphoma with absent monoclonal M protein and CD138+ plasma cells. The final clinicopathologic diagnosis was peritumoral light chain AL amyloidosis, IgM lambda type secondary to high grade (2/3) stage 4 follicular lymphoma. Cardiac MRI was negative for involvement with amyloidosis, and urinalysis didn’t suggest any nephrotic range proteinuria. The patient was started on chemotherapy with anti-CD20 monoclonal antibody (R-CHOP regimen comprising of rituximab, adriamycin, prednisone, vincristine, and cyclophosphamide). He received the treatment for six months, and a follow-up positron emission tomography (PET) scan revealed partial response. The patient was then started on obinutuzumab and bendamustine combination chemotherapy; however, the patient developed anthracycline-induced cardiomyopathy with severe systolic heart failure. The patient could not receive any further chemotherapy and had a fatal outcome secondary to heart failure 18 months after the diagnosis of follicular lymphoma with peritumoral AL amyloidosis.

## Discussion

Light chain (AL) amyloidosis is a rare infiltrative disorder characterized by extracellular deposition of misfolded fibrillar aggregates of monoclonal immunoglobulin light chain protein in a beta-pleated sheet configuration. IgM-related AL amyloidosis (IgM-AL) is a rare variant (6-10%) of AL amyloidosis cases with discrete clinical features [18]. In comparison to non-IgM AL, organ involvement is more frequent in lung and lymph nodes while less common in cardiac and renal tissues [11]. The pattern of amyloid deposition may be widespread to distant organs, i.e., systemic, or restricted to the site of production such as detectable lymphoma, i.e., peritumoral. Considering their rarity, the exact incidence of peritumoral amyloidosis and that associated with IgM-lambda is not known.

AL amyloidosis is an uncommon disorder but a well-recognized complication of lymphoma. Neoplastic B cells as a proliferative center for amyloidogenic Ig light chains has been identified in only about 2% of AL amyloidosis cases [11, 16-18]. This again has been most commonly seen as a systemic pattern of amyloid deposition. The pathophysiology for why in some cases, amyloid deposits distally, while others limit to peritumoral areas has still not been identified. What makes our case rare and unique is the novel presence of the peritumoral distribution of the IgM-AL amyloid in advanced follicular lymphoma with bone marrow involvement. To the best of our knowledge, we discovered two cases of systemic IgM-AL with co-existing follicular lymphoma, which were described in a large case series; however, ours is the first case report describing peritumoral features explicit to them [18]. Those patients had high levels of M protein and multiorgan involvement by amyloid. It is also important to note that there are no cases of transformation from a peritumoral to a systemic syndrome that have been reported suggesting that there are distinct pathophysiologic mechanisms that drive these disease entities.

In reviewing the medical literature, it is clear that peritumoral amyloidosis follows an indolent clinical course, and most patients are reported to remain minimally affected or asymptomatic over prolonged follow-up with little or no treatment [19-20]. This data advocates the implementation of a palliative treatment approach similar to that employed in the management of low-grade lymphomas. Most patients receive alkylator-based therapy with or without rituximab. In lymphoma patients who present with peritumoral amyloidosis, response assessment is typically limited by low or undetectable pretreatment levels of M protein. This is different from the lymphoma patients who present with systemic amyloidosis syndrome who typically have a more aggressive clinical course with a median survival of 11 to 49 months [12,14,18]. Mortality is typically the result of complications related to cardiac and renal involvement.

## Conclusions

In summary, this case report describes the clinicopathologic features of follicular lymphoma associated with peritumoral AL amyloidosis. We aim to add to the existing literature some important descriptive characteristics of this rare disease condition. The underlying biologic mechanisms remain elusive and such association between peritumoral amyloidosis and lymphoproliferative disorders needs to be further explored in order to determine effective treatments and predict clinical outcomes.
